# Mitochondrial transfer: a new perspective on tumor-microenvironment crosstalk driving metastasis

**DOI:** 10.3389/fimmu.2026.1922061

**Published:** 2026-08-17

**Authors:** Yuxuan Duan, Anshu Li, Xun Hu, Zhiyong Xiong, Jian Shi, Keshan Wang

**Affiliations:** 1 Institute of Urology, Union Hospital, Tongji Medical College, Huazhong University of Science and Technology, Wuhan, Hubei, China; 2 Department of Gastrointestinal Surgery, Union Hospital, Tongji Medical College, Huazhong University of Science and Technology, Wuhan, Hubei, China; 3 Department of Urology, The Second Hospital of Huangshi, Huangshi, Hubei, China; 4 Department of Urology, Union Hospital, Tongji Medical College, Huazhong University of Science and Technology, Wuhan, Hubei, China

**Keywords:** cancer cell plasticity, immune evasion, metastasis, mitochondrial transfer, tumor microenvironment

## Abstract

Tumor metastasis is the leading cause of cancer-related deaths. It depends not only on the intrinsic properties of cancer cells but also on their active shaping of the tumor microenvironment. Recent studies have identified mitochondrial transfer(MT) as a key mechanism underlying this tumor–microenvironment crosstalk. Beyond serving as a simple metabolic rescue pathway, MT functions as an intercellular reprogramming process that promotes metastatic progression. In this Review, we propose that MT acts as a tripartite educator during metastasis by driving three coordinated programs: metabolic licensing, immune rewiring, and stromal remodeling. Metabolic licensing enhances the survival and invasive capacity of cancer cells. Immune rewiring helps tumor cells evade immune surveillance. Stromal remodeling reprograms the tumor microenvironment into a permissive niche that supports tumor establishment. We summarize current evidence supporting each of these three programs and discuss emerging therapeutic strategies aimed at either blocking pathological MT or exploiting it for antitumor intervention.

## 1 Introduction

Cancer metastasis is the leading cause of cancer-related deaths, accounting for approximately 80% of all cancer deaths (1). Cancer metastasis transforms a localized tumor into a systemic disease, leading to organ failure by invading vital organs and causing widespread treatment resistance, ultimately resulting in completely uncontrolled disease progression and accelerated death (1, 2).

The shaping of the tumor microenvironment (TME) plays a crucial role in tumor metastasis. Since Stephen Paget first proposed the “seed and soil” hypothesis in 1889, this theory has evolved from a passive selection model into a dynamic, active process in which cancer cells (the seeds) not only adapt to local and distant microenvironments (the soil), but also actively reshape these environments through metabolic reprogramming, immune evasion, and matrix activation, thereby establishing favorable niches for their own establishment and growth (3, 4). This process of dynamic remodeling of the microenvironment essentially relies on the complex and intricate communication network established between cancer cells, stromal cells, and immune cells within TME. Through direct contact, soluble mediators, and the transfer of functional organelles, this network facilitates bidirectional exchange of signals and substances between cells, forming the structural foundation for tumor cells to actively remodel the microenvironment.

Mitochondrial transfer (MT), defined as the intercellular exchange of intact mitochondria or mitochondrial components, has been shown to be a critical mechanism by which tumors communicate with and interact with their microenvironment (5–7). In recent years, MT has increasingly been recognized as a key process in cancer progression and metastasis (8). In 2004, Rustom et al. discovered that tunnel nanotubes (TNTs) in PC12 cells could mediate the direct transport of organelles between cells, challenging the traditional view of cells as “closed entities” and fundamentally transforming our understanding of intercellular communication (9). Since then, extracellular vesicles (EVs), gap junctions, and even cell fusion have been shown to participate in MT across various cancer types. Initially, MT was viewed as a metabolic rescue mechanism (10) —that is, supplying energy to energy-deprived cancer cells by “stealing” functional mitochondria—but it is now recognized as a more complex, multifaceted signaling event that influences cell fate, immune function, and malignant behavior (11–13). For example, even when the energy-producing function of transferred mitochondria is disrupted, they can still initiate immune evasion programs by activating the cyclic GMP-AMP synthase (cGAS)-stimulator of interferon genes (STING) pathway. Conversely, tumor cells can also transfer mitochondria carrying mutated or damaged mitochondrial DNA (mtDNA) to immune cells, impairing their metabolic function and reducing their antitumor activity, thereby subverting immune surveillance (14). This suggests that MT is not merely an energy exchange but a powerful tool for signal remodeling and has become a central mechanism by which cancer cells educate and reshape TME.

Based on this, this review proposes an integrative framework: MT does not merely serve to supply energy to tumors, but rather functions as a form of intercellular communication that facilitates three interconnected programs throughout the metastasis process: (1) metabolic licensing, (2) immune rewiring, and (3) stromal remodeling. We will synthesize the latest research advances on MT in the context of cancer metastasis, focusing on how mitochondrial exchange between different donor and recipient cells synergistically drives metastatic cascades within these three processes. Finally, based on this framework, we will discuss emerging therapeutic strategies that target pathological MT or harness it to enhance antitumor immunity (15).

## 2 Modes of mitochondrial transfer in tumors

### 2.1 Tunneling nanotubes

Among the currently recognized routes of MT, TNTs appear to be one of the most important contact-dependent pathways in tumors (16). By forming long, thin cytoplasmic bridges between adjacent or even relatively distant cells, TNTs enable the direct movement of mitochondria and thereby facilitate metabolic coupling between tumor cells or between tumor cells and stromal cells (17, 18). This transport process is classically mediated by Mitochondrial Rho GTPase 1 (Miro1), which links mitochondria to the intracellular trafficking machinery (19). However, emerging evidence indicates that TNTs-dependent MT is highly heterogeneous across tumor contexts. In some models, such as leukemia, neuroblastoma, and astrocytoma, α-synuclein can preserve TNTs formation and mitochondrial trafficking despite Miro1 deficiency, suggesting that alternative regulatory programs may compensate for canonical transport machinery (20). In addition, proteins involved in intercellular connectivity, including gap junction protein 43 (Cx43) in breast cancer and growth-associated protein 43 (GAP43)-associated tumor microtubule networks in glioma, have also been implicated in TNTs formation or TNTs-related mitochondrial exchange (21, 22). These observations support the view that TNTs function not simply as passive conduits, but as dynamically regulated intercellular communication structures whose molecular control may vary according to tumor type and microenvironmental context.

### 2.2 Extracellular vesicles

Unlike contact-dependent tunneling nanotubes, EVs constitute the principal non-contact pathway for intercellular MT in TME (23). EVs are lipid-bilayer-enclosed particles that carry tetraspanin markers such as CD9, CD63, and CD81 (24, 25), and can be classified into several subtypes based on size and biogenesis (26, 27). As natural intercellular messengers, EVs are well-suited for MT: their lipid bilayer protects encapsulated mitochondrial cargo from extracellular degradation, and surface ligands enable targeted delivery to specific recipient cells (28). Some EVs transport only mitochondrial DNA, whereas others encapsulate intact, functional mitochondria, which are delivered via membrane fusion or endocytosis to restore mitochondrial function and modulate signaling pathways (29, 30). Several molecular regulators have been implicated in this process. In renal cell carcinoma, sorting nexin 9 (SNX9) facilitates mtDNA transport into the cytoplasm prior to vesicle packaging (31). In breast cancer, Metabotropic glutamate receptor 3(mGluR3) activation promotes Ras-related protein Rab-27 (Rab27)-dependent EVs release, and mtDNA packaging depends on PTEN-induced kinase 1 (PINK1) (32). Despite these advances, most mechanistic insights into EVs-mediated MT derive from non-cancer settings, and the regulatory networks governing this process in tumors remain incompletely understood, highlighting a critical area for future investigation.

### 2.3 Adhesion-mediated mitochondrial transfer

Gap junctions (GJs) have traditionally been regarded as channels that mediate the direct exchange of small molecules between adjacent cells (33). However, a unique process known as “gap junction internalization” has been observed in mouse ovarian follicular tissue: when cells reabsorb gap junctions formed between two cells, they simultaneously take up portions of the adjacent cell’s cytoplasm, thereby internalizing large molecular structures such as intact mitochondria or endosomes into their own cytoplasm (34). According to a recent consensus statement on MT nomenclature, this Cx43-dependent contact-mediated transfer mechanism is defined as “adhesion-mediated mitochondrial transfer” (35). In this process, Cx43 serves as an indispensable structural scaffold protein constituting gap junctions. Notably, it has not yet been directly observed in tumor models, and its role in MT within TME remains an open question warranting further investigation.

### 2.4 Cell fusion

First, it should be noted that cell fusion is not classified as a canonical mechanism of MT according to recent consensus (35). Unlike selective endocytosis, cell fusion results in the complete merger of two cells, forming a hybrid cell that permanently shares all cytoplasmic contents (36). Although this process fundamentally reshapes the metabolic profile and malignant potential of the recipient cell (37), it does not in itself constitute MT because there is no distinct donor or recipient cell following fusion. However, given that mitochondrial mixing does occur during fusion, we briefly discuss this here. In 2024, Qiao et al. demonstrated that in adenoid cystic carcinoma, cell fusion depends on transmembrane protein 16F (TMEM16F)-mediated phosphatidylserine externalization, which acts as a fusion-promoting signal (37). Furthermore, an endocytic mechanism has been described in glioblastoma: tumor cells acquire mitochondria by phagocytosing tumor-activated stromal cells (TASCs), although the molecular details of this process remain to be elucidated (38).

**Figure 1:**
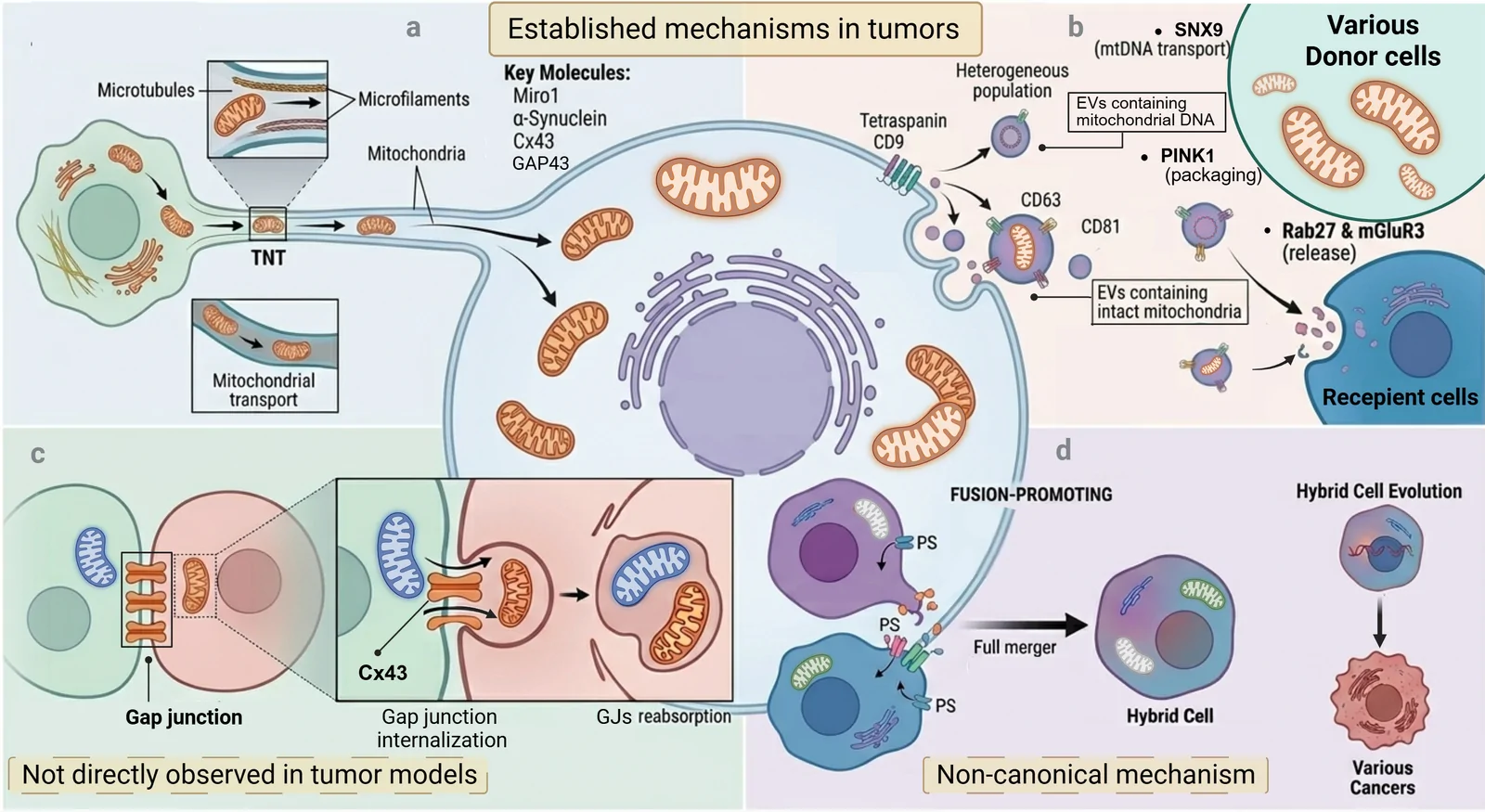
Modes of mitochondrial transfer in tumors. **(a)** Tunneling nanotubes (TNTs). TNTs form solid bridges between cells, facilitating the intercellular transfer of structures such as microtubules and mitochondria. Key regulatory factors include Miro1, α-synuclein, Cx43, and GAP43. **(b)** Extracellular vesicles (EVs). EVs, which express tetra-transmembrane proteins (CD9, CD63, CD81, etc.), mediate contactless mitochondrial transfer (MT) by delivering intact mitochondria or mitochondrial DNA (mtDNA). SNX9 facilitates mtDNA transport, PINK1 packages mtDNA into EVs, and Rab27 and mGluR3 jointly regulate EVs release. **(c)** Adhesion-mediated mitochondrial transfer. Cx43 forms gap junction channels. Through gap junction internalization, cells can take up intact mitochondria from neighboring cells. Although this mechanism has been observed in non-cancerous settings, its role in most tumors remains unclear. **(d)** Cell fusion. Phosphatidylserine (PS) exposure mediated by TMEM16F serves as a signal promoting fusion. Complete cell fusion results in hybrid cells that permanently share all cytoplasmic contents, followed by nuclear genome recombination and post-fusion selection, which can drive the evolution of hybrid cells. Among these, transfer via TNTs and EVs **(a, b)** are established mechanisms supported by direct evidence obtained from various tumor models; adhesion-mediated mitochondrial transfer **(c)** has not yet been directly observed in tumor models and remains a speculative hypothesis; cell fusion **(d)**, due to the absence of clearly identified donor or recipient cells, does not constitute a classic form of MT, but is discussed here as a process contributing to mitochondrial mixing. (Created with BioRender.com).

Among the mechanisms discussed above, TNTs- and EVs-mediated MT are supported by relatively substantial evidence in tumor models, whereas adhesion-mediated MT has not yet been directly observed in tumors and remains a speculative hypothesis at this stage. Cell fusion, although documented in certain tumor contexts, does not constitute a canonical form of MT according to recent consensus (**Figure 1**). Therefore, future investigations into MT in TME should primarily focus on TNTs- and EVs-mediated pathways, while exercising caution when interpreting other proposed mechanisms. In addition to the mechanisms described above, other forms of MT have been reported, such as free mitochondria and mitochondria-derived vesicles; however, most studies have merely confirmed their existence and have not yet fully elucidated the complete transport pathway from donor to recipient, particularly within TME (39, 40).

**Table 1** summarizes the relevant regulatory factors and associated cancer types based on the classification of mechanisms underlying MT. However, a systematic understanding of the general regulatory mechanisms underlying this process is currently lacking. In particular, while Adhesion-mediated MT has been observed in non-cancer settings, it has not yet been directly demonstrated in tumor models, highlighting the need for further investigation to identify context-specific biomarkers for targeted interventions (**Table 1**).

**Table 1:** Mechanisms and regulators of mitochondrial transfer.

Mechanism	Key molecules	Function	Cancer type	References
Tunneling nanotubes (TNTs)	Miro1	Mediates mitochondrial connection to microtubules/actin	Melanoma, Hepatocellular carcinoma	(41, 42)
	α-Synuclein	Promotes TNTs formation and mediates mitochondrial entry into TNTs	Leukemia, Neuroblastoma, Astrocytoma	(20)
	Cx43	Regulates TNTs formation	Breast cancer	(21)
	GAP43	Drives tumor microtubule formation	Glioma	(22)
Extracellular vesicles (EVs)	SNX9	Transports mtDNA to the cytoplasm	Renal cell carcinoma	(31)
	PINK1	Packages mtDNA into EVs	Breast cancer	(32)
	Rab27	Regulates EVs release		
	mGluR3	Promotes EVs release		
Adhesion-mediated mitochondrial transfer	Cx43	Forms gap junction internalization	Not reported in tumors	(34)
Cell fusion	TMEM16F	Triggers cell fusion	Adenoid cystic carcinoma	(37)

Miro1, mitochondrial Rho GTPase 1; Cx43, Connexin 43; GAP43, growth-associated protein 43; SNX9, sorting nexin 9; PINK1, PTEN-induced kinase 1; Rab27, Ras-related protein Rab-27; mGluR3, metabotropic glutamate receptor 3; TMEM16F, transmembrane protein 16F.

## 3 Metabolic basis of mitochondrial transfer in metastasis

Different metabolic patterns confer distinct functions on cells, and tumor metabolism plays a key role in promoting tumor metastasis (43). Before exploring the specific functions of MT, it is necessary to first understand the metabolic characteristics of TME, as these determine why MT can produce such far-reaching functional consequences.

Cancer cells are known for the Warburg effect, in which they preferentially undergo glycolysis even under aerobic conditions, leading to an acidic microenvironment. Although this metabolic reprogramming supports proliferation and invasion, TME is far from metabolically homogeneous (44–47). Circulating tumor cells (CTCs) and metastasis-initiating cells (MICs) rely heavily on mitochondrial oxidative phosphorylation (OXPHOS) to meet their enormous energy demands during dissemination and colonization (48–50). This stands in stark contrast to most primary tumor cells, which rely primarily on glycolysis.

Immune cells also exhibit profound metabolic plasticity. T cells primarily rely on glycolysis during early activation, but their long-term survival and the formation of memory T cells increasingly depend on OXPHOS (51, 52). Importantly, persistent antigen exposure and nutrient deprivation in TME impair T cell mitochondrial integrity, leading to metabolic dysfunction and reduced antitumor activity (53, 54). For macrophages, M1-type macrophages rely on glycolysis and exert antitumor functions, whereas M2-type macrophages rely on OXPHOS and promote tumor growth (55, 56).

Cancer-associated fibroblasts (CAFs), as one of the primary stromal cell types in TME, play a critical role in promoting tumor progression and exhibit multiple functional subtypes. Traditionally, CAFs were thought to rely primarily on glycolysis for metabolism, providing energy support to cancer cells by secreting metabolites such as lactate and pyruvate (57–59). However, recent studies have shown that fibroblasts that have received mitochondria from cancer cells exhibit significantly increased levels of OXPHOS and ATP production, while simultaneously secreting various cytokines, thereby promoting tumor proliferation and migration (60).

**Figure 2:**
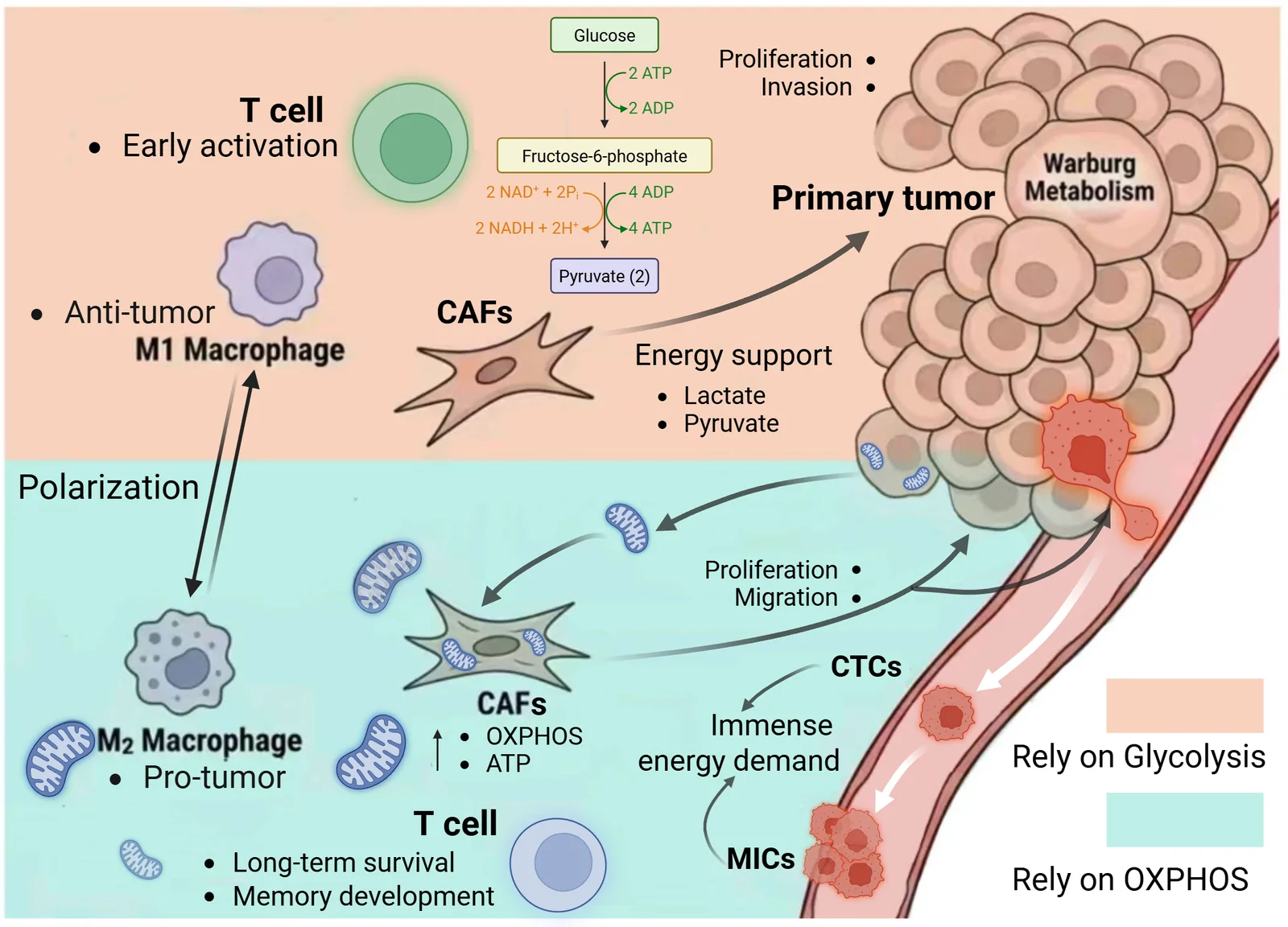
Metabolic basis of mitochondrial transfer in metastasis. Cancer cells exhibit the Warburg effect (i.e., glycolysis under aerobic conditions), which provides the energy required for their proliferation and invasion. However, circulating tumor cells (CTCs) and metastasis-initiating cells (MICs) face enormous energy demands during migration and colonization and therefore rely more heavily on oxidative phosphorylation (OXPHOS). In immune cells, T cells rely primarily on glycolysis during early activation, whereas the formation and long-term survival of their memory cells depend on OXPHOS. M1 macrophages primarily rely on glycolysis to exert antitumor effects, while M2 macrophages rely on OXPHOS and exhibit pro-tumor properties. Traditionally, cancer-associated fibroblasts (CAFs) have been known to support cancer cells by secreting lactate and pyruvate, a process that relies on glycolysis; however, upon receiving mitochondria from cancer cells, CAFs can also switch to OXPHOS, thereby promoting tumor cell proliferation and migration. These starkly different metabolic preferences confer specific functions on different cells and lay the foundation for mitochondrial transfer to play a key intercellular role in the metastasis process. (Created with BioRender.com).

These metabolic differences provide a crucial foundation for understanding how MT regulates cellular function by reshaping the energy and metabolic states of both donor and recipient cells. In this context, MT acts as a core player in the tumor metastasis process, collectively driving metastasis through three interconnected pathways (**Figure 2**).

## 4 Mitochondrial transfer as a tripartite educator in metastasis

### 4.1 Metabolic licensing

During migration, cancer cells face challenges such as energy deprivation, oxidative stress, and blood flow shear stress. In this context, MT serves as a direct means of metabolic support mechanism, enhancing the metabolic plasticity and stress resistance of cancer cells by delivering functional mitochondria to them.

Multiple stromal cell types have been identified as sources of mitochondria. Hoover et al. found that neurons can directly transfer functional mitochondria to tumor cells. Cancer cells that acquire mitochondria exhibit significantly higher survival rates than ordinary cancer cells when exposed to simulated blood flow shear stress and oxidative stress, providing critical support for cancer cell entry into the circulatory system (61, 62). In breast cancer and chronic lymphocytic leukemia, platelet-derived MT increases ATP levels and induces cells to adopt a more invasive migratory phenotype (63, 64). Adipose-derived stem cells can supply mitochondria to cancer cells via TNTs and GJs (65). Glioblastoma cells can acquire mitochondria from astrocytes (22). Hepatocellular carcinoma cells can obtain functional mitochondria from neighboring hepatic stellate cells (HSCs) via TNTs, and they also transport damaged mitochondria to surrounding macrophages via EVs for clearance, thereby promoting tumor progression (15). When THY1^+^ cancer stem cells (CSCs) encounter neutrophils, they not only induce the release of damaged mitochondria rich in reactive oxygen species (ROS) from the neutrophils but also, through the overexpression of THY1, directly enhance the CSCs’ own phagocytic capacity, enabling them to efficiently uptake these released mitochondria. Mitochondrial reactive oxygen species (mtROS) transferred into CSCs inhibit the activity of prolyl hydroxylase (PHD), thereby blocking the degradation of hypoxia-inducible factor 1-alpha (HIF-1α) and establishing a pseudo-hypoxic state that helps cancer cells acquire metabolic plasticity and anti-apoptotic capabilities, among other effects (66).

In addition, MT also occurs between tumor cells. In breast cancer cells, mtDNA-containing EVs derived from PINK1-mediated packaging have been shown to activate Toll-like receptor 9-mediated signaling in recipient cells, driving migratory and invasive phenotypes (32). In hepatocellular carcinoma (HCC), HMGB1 acts as a key signaling molecule in the hypoxic microenvironment. By upregulating the mitochondrial transport protein RHOT1 and the protein RAC1, which is formed by TNTs, it promotes the transfer of mitochondria from highly invasive HCC cells to less invasive cells, significantly enhancing their migratory and invasive capabilities and ultimately synergistically promoting tumor metastasis (42, 67). A similar effect has been observed in the co-culture of two bladder cancer cell lines, where T24 cells are more invasive than RT4 cells. When mitochondria are transferred from T24 to RT4, RT4 exhibits a more invasive phenotype (68).

The direct transfer of intact organelles, such as mitochondria, provides cancer cells with a more direct and thorough means of metabolic reprogramming. Cancer cells that were previously energy-deprived and struggled to survive gain access to ample energy, enabling them to survive in the circulatory system and acquire enhanced migratory and invasive capabilities, thereby successfully initiating the metastatic cascade. These observations highlight a central role of MT in supporting the metabolic fitness and invasive capacity of cancer cells during the phases of metastasis.

### 4.2 Immune rewiring

Beyond its role in metabolic support, MT also functions as a signaling mechanism that remodels immune cell function. By suppressing anti-tumor immunity, it helps cancer cells evade immune surveillance and facilitates metastatic dissemination and establishment.

In a recent study, Ikeda et al. found that tumor cells can transfer mutated mtDNA to CD8^+^ T cells and transport the deubiquitinating enzyme ubiquitin-specific peptidase 30 (USP30) to the surface of T cell mitochondria. By removing ubiquitin tags from the mitochondrial surface, USP30 allows damaged mitochondria to evade autophagy-mediated clearance, leading to the accumulation of “mitochondrial waste” within T cells, which in turn causes defects in T-cell effector function and memory formation (14).

In another study, Terasaki et al. found that tumor cells impair the antigen-presenting and cytotoxic functions of immune cells by hijacking their mitochondria. Furthermore, after fusing with the cancer cells’ own mitochondria, these hijacked mitochondria can trigger a type I interferon response and immune evasion programs by activating the cGAS-STING pathway, ultimately promoting tumor cell metastasis to lymph nodes (69). Notably, the team also found that mitochondria actively acquired by cancer cells from stromal cells were unable to fuse; consequently, they could not activate signaling pathways or promote metastasis (69).

In addition to the donor identity of the mitochondria, whether the transfer is active or passive is also a key factor determining the outcome. In the context of bone metastasis, when tumor cells passively acquire mitochondria from bone cells, the immune system is actually able to exert an antitumor effect (70).

These findings indicate that the ultimate effect of MT is not simply “acquisition equals oncogenicity,” but rather depends on the identity of the donor and recipient, as well as whether the transfer is active or passive. The function of MT has expanded from a simple “energy rescue” mechanism to a complex “signal remodeling” process, the outcome of which is regulated by the specific cellular microenvironment. It should be noted that it remains unclear whether all mitochondria from immune cells actively hijacked by cancer cells promote metastasis; further research is needed to elucidate this.

Moreover, cells within TME shape the immunosuppressive microenvironment through direct or indirect mechanisms. Senescent cells actively encapsulate mtDNA within EVs and release them into polymorphonuclear myeloid-derived suppressor cells (PMN-MDSCs), which strongly inhibit lymphocyte cytotoxic function; this activates the cGAS-STING-nuclear factor kappa-light-chain-enhancer of activated B cells (NF-κB) signaling axis, thereby enhancing their immunosuppressive activity (71). Kuo et al. found in a xenograft animal model that mitochondria from endothelial cells to melanoma cells activate the nuclear factor erythroid 2-related factor 2 (Nrf2)/heme oxygenase-1 (HO-1) pathway, which can reprogram macrophages from the antitumor M1 phenotype to the pro-tumor M2 phenotype by upregulating transforming growth factor-beta (TGF-β)1 secretion, thereby creating an immunosuppressive microenvironment (72).

It is worth noting that, to date, no studies in the field of oncology have reported on the direct involvement of regulatory T cells (Tregs) in MT. Currently, evidence is only available from other disease models. For example, a study on multiple sclerosis demonstrated that mesenchymal stem cells (MSCs) can transfer mitochondria to CD4+ T cells, thereby inhibiting the glycolytic metabolism of Th17 cells and enhancing oxidative phosphorylation. This process suppresses IL-17 production and promotes the conversion of Th17 cells into Treg cells (73). Studies on inflammatory responses have similarly shown that MSCs can directly reprogram T cells by transferring mitochondria to CD4+ T cells, inducing their differentiation into highly suppressive Treg cells, thereby limiting inflammatory responses both *in vivo* and *in vitro* (74). This suggests a possibility that has not yet been explored: whether tumor cells in TME actively modulate MSCs to induce Treg cell generation through MT, thereby promoting the formation of an immunosuppressive microenvironment. This hypothesis requires direct experimental validation in the context of TME.

### 4.3 Stromal remodeling

Stromal remodeling represents the third dimension of this framework. Through MT, tumor cells reprogram the stromal microenvironment into a state that promotes tumor progression. This process occurs not only at the primary tumor site but also extends to distant sites via systemic signaling, paving the way for metastatic colonization.

Fibroblasts are among the most abundant stromal cells in TME, and their transformation into CAFs is a central event in stromal remodeling and the promotion of tumor metastasis (75). In various tumor models, including skin cancer, breast cancer, and pancreatic cancer, it has been observed that tumor cells transfer their own mitochondria to neighboring fibroblasts via MIRO2-mediated TNTs, inducing metabolic reprogramming in fibroblasts, primarily manifested by upregulation of OXPHOS and increased ATP production. This metabolic shift directly promotes fibroblast proliferation, extracellular matrix production, and protein secretion, conferring a typical CAF phenotype (60). In addition to intact mitochondria, mitochondrial-derived signaling molecules can also drive this process. Mitochondrial double-stranded RNA (dsRNA) derived from B-ALL cells, upon uptake by MSCs, drives CAF differentiation (6). These CAFs co-evolve with the disease, altering the biochemical and physical structure of TME, thereby modifying the behavior of surrounding stromal cells and cancer cells (76). Spatially, they can generate topographic cues that guide cancer cell migration (77); metabolically, CAFs provide metabolic fuel such as glutamine and pyruvate through autophagy (51, 78), enabling cancer cells to maintain growth even within the nutritionally deprived TME; and in terms of signaling, CAFs secrete large amounts of cytokines (such as TGF-β and IL-6) to promote cancer cell invasion and immune suppression (79).

Importantly, CAFs are not only products of stromal remodeling; they can also function as part of a metabolic licensing mechanism, utilizing MT to support tumor metastasis. CAFs transfer mitochondria to triple-negative breast cancer cells via TNTs, significantly enhancing their mitochondrial ATP production and 3D migration capacity (80). In both *in vitro* and *in vivo* prostate cancer models, cancer cells have been observed to hijack CAF mitochondria by forming cell bridges, thereby promoting malignant progression (81). Qiao et al. found that in adenoid cystic carcinoma, cancer cells chemotactically attract CAFs to their vicinity. Subsequently, CAFs expose signals via TMEM16F-mediated phosphatidylserine externalization, triggering the formation of nanotunnels and cell fusion, thereby restoring cancer cell metabolic function through MT (37). Zhou et al. found in lung cancer that highly metastatic tumor cells reprogram normal fibroblasts into inducible cancer-associated fibroblasts (iCAFs) via miR-1290. Subsequently, iCAFs release mtDNA into tumor cells with mitochondrial dysfunction, helping them restore mitochondrial function and resistance to oxidative stress, ultimately promoting tumor metastasis (82).

The spatial extent of stromal remodeling is not limited to the immediate vicinity of the primary tumor. The formation of a pre-metastatic niche is one of the most groundbreaking discoveries of the past two decades (83). Even before cancer cells begin to metastasize, the primary tumor can secrete factors that induce the formation of a specialized microenvironment suitable for the future survival of cancer cells (84–86). However, direct evidence linking the pre-metastatic niche to MT remains scarce at this stage. Only a recent study by Guan et al. found that MT is also involved in the formation of the pre-metastatic niche in colorectal cancer. Tumor-derived EVs carry intact circular mtDNA and transfer it to adjacent normal colonic epithelial cells (CECs). This transfer activates the mitochondrial respiratory chain in CECs, induces ROS-driven RelA nuclear translocation, and upregulates TGF-β1 expression via the NF-κB pathway, thereby remodeling distant normal tissues through TGF-β/Smad signaling to prepare for cancer cell colonization and growth (87).

**Figure 3:**
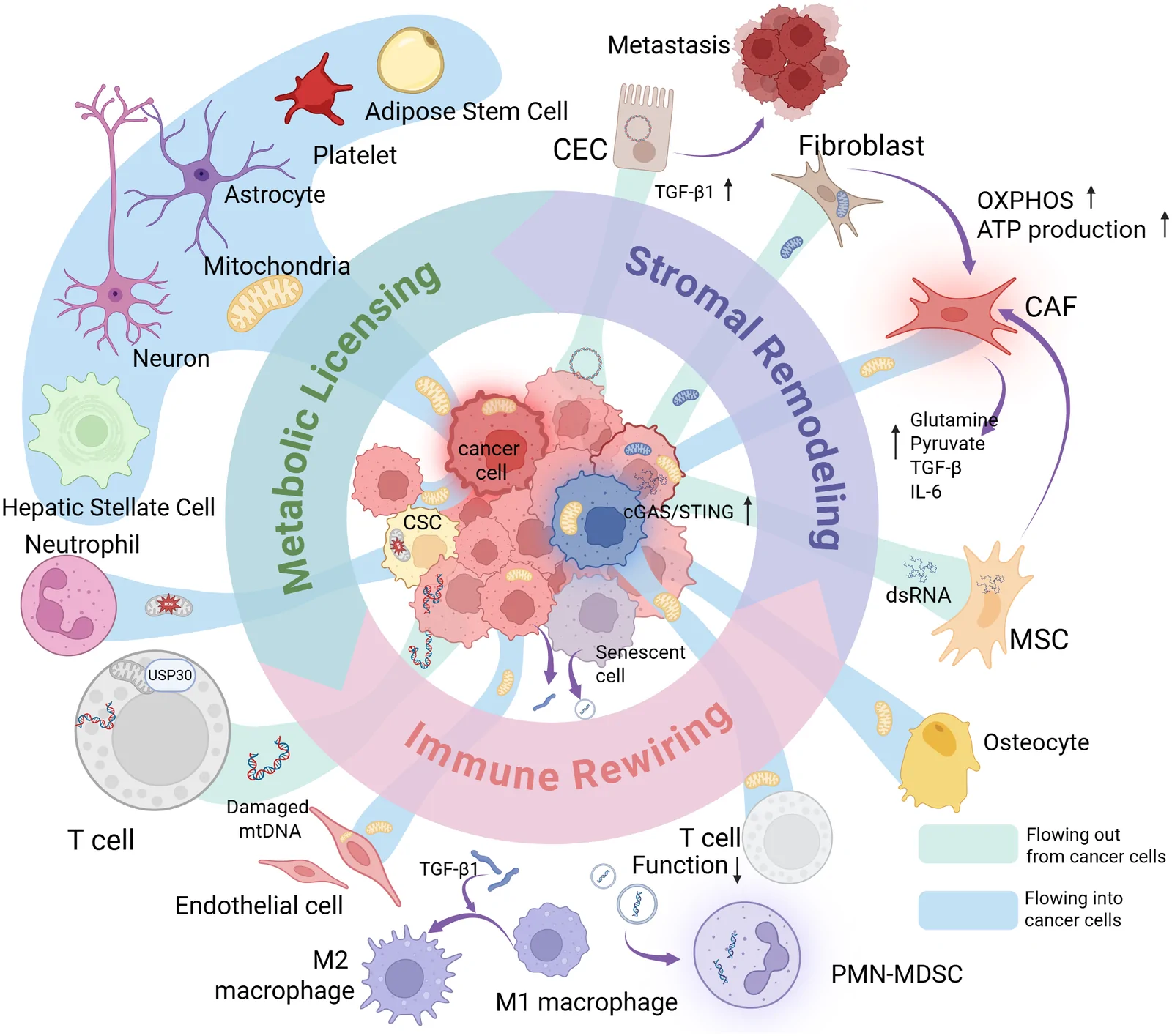
Mitochondrial transfer acts as a tripartite educator to drive the metastatic cascade. Metabolic licensing refers to the process by which cancer cells acquire functional mitochondria from cells such as neurons and platelets, thereby enhancing their metabolic plasticity, stress resistance, and invasive capacity, and providing the energy foundation for the initiation of metastasis. Immune rewiring manifests as cancer cells actively weakening immune cells by hijacking their mitochondria, activating pro-metastatic pathways, and exporting mitochondrial components to shape an immunosuppressive microenvironment. However, mitochondrial transfer(MT) is not invariably pro-tumorigenic; in the context of bone metastasis, passive acquisition of mitochondria from osteocytes can instead activate anti-tumor immunity, illustrating the context-dependent duality of mitochondrial transfer in immune modulation. Stromal remodeling occurs when cancer cells reprogram fibroblasts into tumor-associated fibroblasts via MT, thereby establishing a pro-tumor niche; these fibroblasts can also transport mitochondria in the opposite direction to drive tumor progression, and this remodeling can extend to distant sites to form pre-metastatic niches. These three processes synergistically promote tumor metastasis. (Created with BioRender.com).

In summary, MT is not a single metabolic rescue event in the process of tumor metastasis, but rather involves bidirectional exchange that systematically modulates TME through three distinct pathways, thereby synergistically driving the metastatic cascade(**Figure 3**). A structured summary of the experimental evidence supporting these three programs, including donor-recipient pairs, transfer routes, cargo types, biological outcomes, and levels of evidence, is provided (**Supplementary Table 1**).

## 5 Therapeutic implications

**Figure 4:**
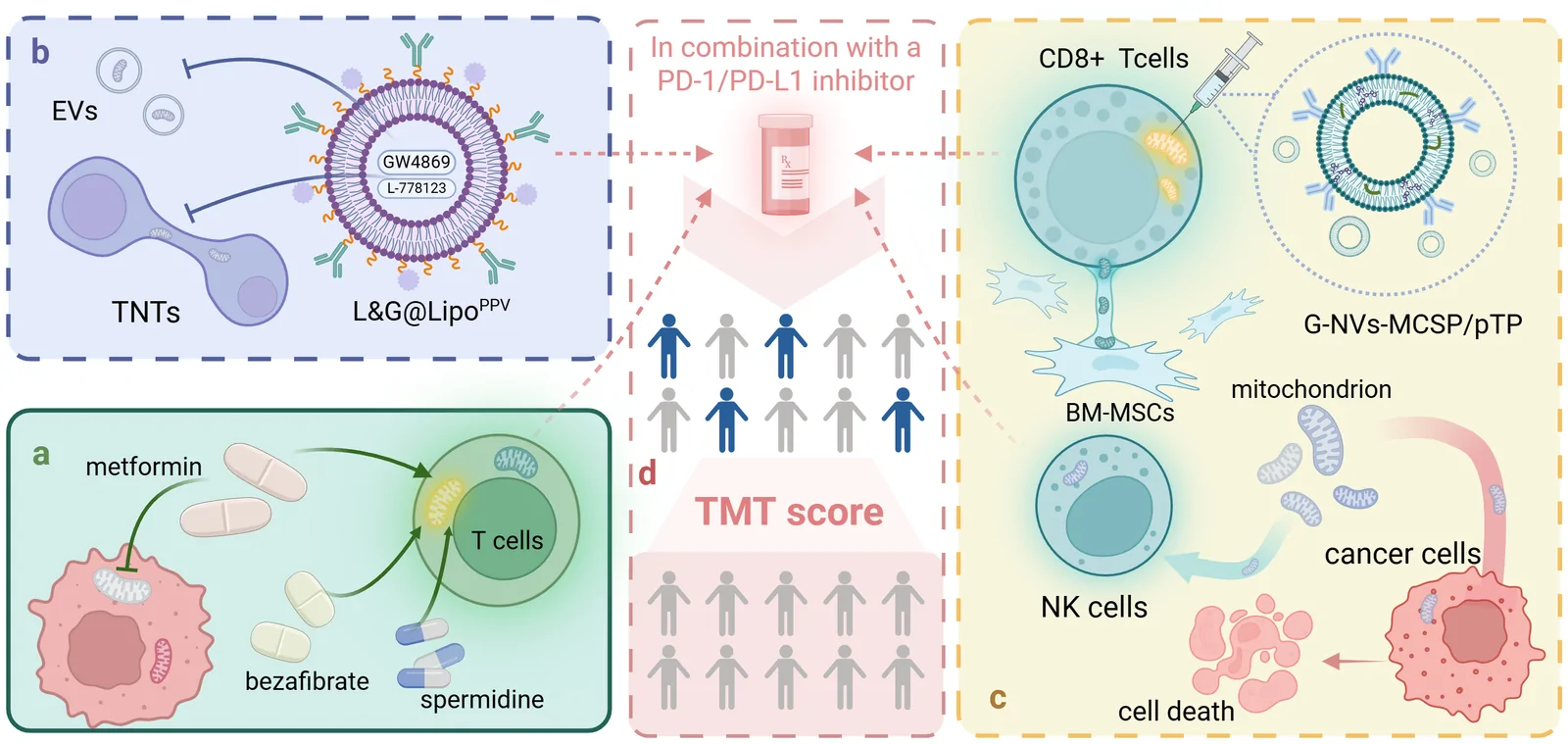
A multilayered hypothetical therapeutic framework targeting mitochondrial transfer. This figure illustrates a conceptual framework for therapeutic strategies centered on mitochondrial transfer(MT). Although these strategies hold promise for combination with immune checkpoint inhibitors and may eventually benefit patients with high Tumor Mitochondrial Transfer (TMT) scores, this framework remains a long way from actual clinical application. The solid-line boxes **(a)** represent strategies that have entered clinical trials, but their direct regulatory effects on MT in cancer patients have not yet been validated; the dashed-line boxes **(b–d)** and dashed arrows represent hypothetical, preclinical, or context-dependent strategies that require further mechanistic and translational research. **(a)** Metabolic modulation as an indirect intervention strategy. On the one hand, it enhances the mitochondrial function of immune cells to boost their antitumor activity; on the other hand, it inhibits mitochondrial metabolism in cancer cells, thereby undermining their survival advantage. **(b)** Blocking pathological mitochondrial transfer to enhance anticancer therapy. GW4869 and L-778123 can block the formation of extracellular vesicles(EVs) and tunneling nanotubes(TNTs), respectively, while increasing tumor sensitivity to immune checkpoint inhibitors. The L&G@Lipo^ppv^ nanocarrier simultaneously disrupts both pathways. **(c)** Harnessing mitochondrial transfer for therapeutic benefit. Transferring healthy mitochondria to T cells and NK cells enhances their metabolic adaptability and antitumor efficacy; utilizing the G-NVs-MCSP/pTP system for precise delivery protects and repairs mitochondria. **(d)** Patient stratification and translational outlook. Patients with high TMT scores may derive greater therapeutic benefit, and the TMT score can bridge the gap between mechanism discovery and translational application. (Created with BioRender.com).

Viewing MT as a central mechanism of tumor-microenvironment communication opens up new therapeutic possibilities for controlling metastasis. From a translational perspective, MT-related strategies can be broadly categorized into two types: blocking pathological mitochondrial exchange or leveraging MT to achieve therapeutic benefits. However, most of the strategies discussed in this section remain in the preclinical or early experimental stages, are largely adapted from other disease models, and face challenges such as delivery specificity and off-target toxicity (88, 89). Even drugs such as metformin, Bezafibrate, and spermidine, which have been studied in other clinical contexts, have not yet been validated for MT-related cancer indications. Therefore, this therapeutic framework should be viewed as a conceptual roadmap rather than a clinically actionable pathway. With this caveat in mind, the following sections will discuss the current evidence and translational prospects for various MT-targeting strategies. Nonetheless, these proof-of-concept investigations provide valuable insights for developing more selective and clinically viable MT-targeting strategies (**Figure 4**).

### 5.1 Metabolic modulation as an indirect intervention strategy

One therapeutic entry point is to modulate the metabolic consequences associated with MT. Because mitochondrial exchange can enhance OXPHOS, stress tolerance, and immune dysfunction, drugs targeting cellular metabolism may partially counteract its downstream effects. Metformin has demonstrated dual benefits in animal models: on the one hand, it reduces tumor oxygen consumption and alleviates microenvironmental hypoxia by inhibiting the mitochondrial complex I in cancer cells; on the other hand, it enhances antitumor immunity by activating AMP-activated protein kinase in T cells (90, 91). Although clinical trials combining metformin with chemotherapy or immunotherapy are ongoing, no studies have yet reported its direct regulation of tumor mitochondrial metabolism in patients. Similarly, Bezafibrate and other drugs that enhance mitochondrial adaptability have been explored in preclinical studies to improve T-cell function and enhance responses to immune checkpoint inhibitor therapies (92, 93). Some studies suggest that spermidine can also restore mitochondrial function and revitalize senescent T cells (94); however, as a tumor metabolite, it exhibits tumor-promoting effects in glioblastoma models (95), and the specific mechanisms of its interaction with mitochondria remain to be further elucidated. It should be noted that the above strategies primarily target the metabolic outputs of MT rather than the transfer process itself, and therefore may be better viewed as indirect interventions than as specific MT-targeted therapies. Moreover, given that these agents modulate systemic metabolism broadly, their clinical application will require careful patient selection and rigorous monitoring of unintended metabolic effects in non-tumor tissues.

### 5.2 Blocking pathological mitochondrial transfer to enhance anticancer therapy

A more direct therapeutic strategy is to interfere with the transfer process itself, particularly in settings where mitochondrial exchange fuels metastatic progression or suppresses antitumor immunity. Among the currently recognized transfer routes, TNTs, EVs, and Cx43-associated intercellular connections have attracted the greatest translational interest. Although tunnel nanotube formation inhibitors such as cytokinin and lachunculin have completed proof-of-concept studies, they cause widespread disruption of the actin cytoskeleton (5, 14, 96, 97). Therefore, highly selective inhibitors hold greater research value: L-778123 can partially inhibit the formation of pathological tunnel nanotubes without causing global disruption of actin; In animal models, this drug, when combined with a programmed cell death protein 1 (PD-1) inhibitor, significantly reduces mitochondrial translocation and enhances antitumor efficacy (98). However, L-778123’s clinical development was halted due to cardiotoxicity and lack of KRAS inhibition efficacy; it now serves only as a research tool, and its clinical translation remains distant (88, 89). Likewise, inhibition of EVs-mediated MT with GW4869 restored T-cell function and sensitized tumors to PD-1 blockade by preventing the delivery of dysfunctional mitochondria and mutated mtDNA into tumor-infiltrating T cells (14). In addition, nanocarrier-based systems capable of co-delivering inhibitors of both TNTs- and EVs-mediated transfer suggest that simultaneous blockade of multiple MT routes may provide a more effective strategy for suppressing tumor growth and metastasis (15). Cx43 is another promising target because of its role in both TNTs- and gap junction-related intercellular communication. Although Cx43-directed agents such as meclofenamic acid and Cx43 antibody-conjugated nanogels have already shown antitumor activity in specific settings, whether their therapeutic benefit is directly attributable to inhibition of MT remains to be fully clarified (99, 100). Collectively, these preclinical studies support the concept that blocking pathological MT can inhibit tumor progression and enhance immunotherapy responses (45, 101). However, the agents currently available, including L-778123, GW4869, meclofenamic acid, and Cx43 antibody-conjugated nanogels, remain limited by broad-spectrum mechanisms and modest specificity, and none have been clinically evaluated for MT-related indications. Their utility is therefore largely restricted to serving as research tools for mechanistic studies, with clinical translation remaining a distant prospect.

### 5.3 Harnessing mitochondrial transfer for therapeutic benefit

In contrast to strategies aimed at inhibiting pathological transfer, a second emerging direction is to exploit MT for therapeutic gain. Mitochondrial transplantation therapy was initially developed as an approach to restore bioenergetic function in injured tissues, but it is now being explored in oncology in a more nuanced manner (102–105). In some settings, the delivery of healthy mitochondria into tumor cells may reactivate apoptotic programs, alter redox homeostasis, and increase sensitivity to chemotherapy or radiotherapy (106–108). At the same time, MT can also be used to enhance the function of antitumor immune cells. Studies have shown that transplanting healthy mitochondria into CD8^+^ T cells or NK cells can improve their respiratory capacity, proliferative capacity, and cytotoxic activity. Specifically, bone marrow-derived mesenchymal stem cells (BM-MSCs) have been shown to transfer healthy mitochondria to CD8^+^ T cells via TNTs, a process requiring Talin 2 on both donor and recipient cells. Mitochondria-empowered T cells exhibit enhanced expansion and superior antitumor efficacy in tumor-bearing models, leading to prolonged survival (7, 109). In addition, an *in vitro* study demonstrated that healthy mitochondria derived from WRL-68 cells can be transferred to NK-92MI cells, enhancing their proliferative capacity and cytotoxicity against various cancer cell lines, including K562 leukemia cells and several solid tumor cell lines (110). Although these findings remain largely at the preclinical stage, they raise the possibility of metabolically reinforced cell therapies, including next-generation CAR-T or CAR-NK platforms. Beyond direct transplantation, engineered systems such as mitochondrial hitchhiking platforms suggest that the endogenous transport properties of mitochondria can also be used for deep-tissue drug delivery. For example, the engineered biomimetic drug delivery system G-NVs-MCSP/pTP, based on ginseng-derived nanovesicles, is capable of precisely delivering drugs that protect or repair mitochondria to immune cells, and has demonstrated significant antitumor effects and good biosafety in various melanoma models (111). These approaches expand the concept of MT-based therapy from simple replacement of damaged organelles to the broader engineering of intercellular bioenergetic and signaling networks.

Although the results of preclinical studies are encouraging, the clinical translation of Mitochondria transfer and transplantation(MTT) -based therapeutic strategies still faces several key challenges, and most of the current evidence comes from models of non-cancer diseases (112). First, isolated mitochondria have a short lifespan, significantly losing respiratory function after approximately 2 hours, which imposes stringent temporal constraints on clinical application (113). Second, since mitochondria are recognized by the immune system as foreign entities, immune responses may compromise therapeutic efficacy (114). Third, only a small proportion (approximately 10%) of injected mitochondria reach target cells, and the transfer lacks specificity for target cells (115). Non-viable or damaged mitochondria can release damage-associated molecular patterns that activate the immune system (116). Even upon reaching target cells, mitochondria must avoid lysosomal degradation and successfully integrate into the recipient cell’s mitochondrial network (117). In summary, current MTT approaches face major hurdles, ranging from poor delivery efficiency to target cells and immunogenicity, to intracellular integration barriers. These limitations underscore that MTT remains largely at the preclinical stage, and substantial technological innovation is required before it can be translated into clinical practice, particularly in the context of cancer therapy.

### 5.4 Patient stratification and translational outlook

An important remaining challenge is to identify which patients are most likely to benefit from MT-related interventions. In this regard, the development of the Tumor Mitochondrial Transfer (TMT) score provides a potentially valuable framework for patient stratification (118). By quantifying the extent of mitochondrial hijacking from T cells by cancer cells, this score links MT activity to T-cell exhaustion, tumor aggressiveness, and clinical outcome. However, despite its promise, the TMT score remains a research tool at this stage, and its clinical utility requires prospective validation in large, well-designed patient cohorts before it can inform treatment decisions.

## 6 Discussion

Tumor metastasis, the leading cause of cancer-related mortality, depends heavily on the intricate dynamic interactions between cancer cells and their surrounding microenvironment (1). In recent years, the classic “seed and soil” hypothesis has evolved from a passive selection model into an active process (3). Cancer cells reprogram surrounding stromal and immune cells to establish a malignant microenvironment that supports metastasis, colonization, and growth. MT, as a recently identified key mechanism, is emerging as a crucial means by which cancer cells educate the microenvironment. Through multidimensional pathways, including metabolic remodeling, immune evasion, and stromal activation, it systematically constructs a metastasis-promoting ecosystem. It is worth noting that the three-part framework is proposed as an organizing heuristic, rather than as a strictly proven temporal sequence. These processes may overlap, occur simultaneously, or vary depending on tumor type, disease stage, donor cell, recipient cell, and microenvironmental conditions.

This review emphasizes that the function of MT extends far beyond simply providing energy to cancer cells. Acting as a multilevel signaling hub, it reshapes cellular identity and enhances malignant phenotypes by regulating key signaling pathways (such as the cGAS-STING, Nrf2/HO-1, and HIF-1α pathways), and establishes a bidirectional communication network between tumor cells and cells in the microenvironment, coordinating the formation of a metastasis-promoting ecosystem (66, 69, 82). Specifically, the ultimate effects of MT are highly dependent on the identity of the donor and recipient, the mode of transfer, and the spatiotemporal context: when cancer cells acquire mitochondria from immune cells, even if their energy-generating function is compromised, they can still initiate immune evasion programs by inducing type I interferon responses, thereby promoting lymph node metastasis (69); whereas acquiring mitochondria from ordinary stromal cells primarily enhances metabolic adaptability (61, 80). Furthermore, tumor cells can also export mitochondria carrying mutated mtDNA to immune cells, impairing their metabolic function and subverting immune surveillance (14), or induce fibroblasts to acquire a CAFs phenotype through MT, thereby reshaping the stromal microenvironment (60, 82). It is worth noting that MT does not always promote tumor progression; when tumor cells passively acquire mitochondria from osteocytes, this can instead activate an antitumor immune response via the STING pathway, thereby exerting a tumor-suppressing effect (70). The specific effects described above, which are determined by the types of donor and recipient cells, indicate that MT has long since transcended simple metabolic rescue and evolved into a complex signaling reprogramming event. Its ultimate outcome is highly dependent on the specific TME and the identity of the participating cells, and it possesses both pro-tumor and anti-tumor potential.

Accurately defining the biological significance of MT depends first and foremost on rigorous methodological validation of its authenticity; however, current technical approaches for demonstrating true functional MT still face numerous challenges. Although fluorescent dyes such as MitoTracker are widely used, their specificity is limited because they rely on membrane potential for enrichment; furthermore, once the dye leaks out, it can be taken up by neighboring cells, leading to a large number of false-positive signals. Therefore, using these dyes alone as a validation method is not recommended (35, 119). EVs preparations are inherently highly heterogeneous, with significant differences in size and content among different subpopulations. The detection of mitochondrial DNA or proteins in EVs does not equate to the functional transfer of intact mitochondria; therefore, it is essential to clearly distinguish between intact mtDNA and fragmented mtDNA in research (120, 121). Furthermore, cell fusion or the phagocytosis of cellular debris may be misinterpreted as selective organelle transfer, yet both are fundamentally distinct from active, functional MT (35). These methodological pitfalls suggest that a single technical approach is insufficient to confirm the authenticity of MT. Future studies should adopt a multimodal, orthogonal validation strategy, combining live-cell time-lapse imaging, genetically encoded mitochondrial reporter proteins, mtDNA haplotype analysis, the pH-sensitive reporter gene mito-Keima, and transmission electron microscopy, supplemented by functional metabolic assays (such as respiratory chain complex activity, ATP production capacity, or membrane potential recovery assays), to simultaneously validate both the transfer events and the structural and functional integrity of the acquired mitochondria, thereby laying a solid foundation for subsequent mechanistic exploration and therapeutic translation (118, 119, 122, 123).

Based on an understanding of the aforementioned mechanisms, therapeutic strategies targeting MT are evolving from single-intervention approaches toward the development of multi-level, multidimensional systems. On the one hand, blocking pathological MT at its source—for example, by inhibiting MT mediated by tunnel nanotubes or extracellular vesicles—and combining this with immunotherapy (98) holds promise for restoring immune surveillance and overcoming treatment resistance. On the other hand, inspired by the phenomenon of MT, strategies that actively harness its natural transport capacity—such as transplanting healthy mitochondria into immune cells to enhance their metabolic adaptability and antitumor efficacy (109, 110), or constructing “mitochondrial hitchhiking” drug delivery platforms to achieve precise delivery to deep tissues (124) —offer new insights for next-generation cell therapy. Furthermore, the establishment of the TMT score enables the precise identification of patients with high MT activity, laying the foundation for patient stratification in the clinical translation of the aforementioned intervention strategies (118). From patient screening to targeted intervention, precision therapy for MT is gradually forming a complete closed-loop system.

Although this Review highlights MT as a tripartite educator that drives metabolic licensing, immune rewiring, and stromal remodeling during metastasis, this framework still requires rigorous validation across tumor types and disease stages. Future studies should clarify how donor identity, recipient state, transfer route, and microenvironmental stress collectively shape the functional consequences of mitochondrial exchange, and whether these processes can be quantitatively captured in clinical specimens. Equally important, therapeutic targeting of MT must achieve sufficient specificity to disrupt pathological communication without compromising physiological mitochondrial exchange in normal tissues. Thus, the next phase of this field will depend on integrating mechanistic studies, *in vivo* tracking technologies, and clinically annotated datasets to determine whether MT is not only a conceptual advance in tumor biology, but also a tractable target for precision anti-metastatic therapy.

## Glossary

BM-MSCs: Bone Marrow-Derived Mesenchymal Stem Cells
CAFs: Cancer-Associated Fibroblasts
CAR-NK: Chimeric Antigen Receptor Natural Killer Cells
CAR-T: Chimeric Antigen Receptor T-Cell
cGAS: Cyclic GMP-AMP Synthase
CTCs: Circulating Tumor Cells
Cx43: Connexin 43
dsRNA: Double-Stranded RNA
EVs: Extracellular Vesicles
GAP43: Growth-Associated Protein 43
GJs: Gap Junctions
HCC: Hepatocellular Carcinoma
HIF-1α: Hypoxia-Inducible Factor 1-Alpha
HO-1: Heme Oxygenase-1
HSCs: Hepatic Stellate Cells
iCAFs: Inducible Cancer-Associated Fibroblasts
mGluR3: Metabotropic Glutamate Receptor 3
MICs: Metastasis-Initiating Cells
Miro1: Mitochondrial Rho GTPase 1
MSCs: Mesenchymal Stem Cells
MT: Mitochondrial Transfer
mtDNA: Mitochondrial DNA
mtROS: Mitochondrial Reactive Oxygen Species
NF-κB: Nuclear Factor Kappa-Light-Chain-Enhancer of Activated B Cells
NK cells: Natural Killer Cells
Nrf2: Nuclear Factor Erythroid 2-Related Factor 2
OXPHOS: Oxidative Phosphorylation
PD-1: Programmed Cell Death Protein 1
PHD: Prolyl Hydroxylase
PINK1: PTEN-Induced Kinase 1
PMN-MDSCs: Polymorphonuclear Myeloid-Derived Suppressor Cells
PS: Phosphatidylserine
Rab27: Ras-Related Protein Rab-27
ROS: Reactive Oxygen Species
SNX9: Sorting Nexin 9
STING: Stimulator of Interferon Genes
TASCs: Tumor-Activated Stromal Cells
TGF-β: Transforming Growth Factor Beta
THY1: Thy-1 Cell Surface Antigen
TME: Tumor Microenvironment
TMEM16F: Transmembrane Protein 16F
TMT: Tumor Mitochondrial Transfer
TNTs: Tunneling Nanotubes
Tregs: Regulatory T Cells
USP30: Ubiquitin-Specific Peptidase 30
