# Supplementary material for: Mitochondrial transfer: a new perspective on tumor-microenvironment crosstalk driving metastasis
Source: Front Immunol. 2026 Aug 17;17:1922061. doi: 10.3389/fimmu.2026.1922061 (PMC13527376; doi:10.3389/fimmu.2026.1922061)
Supplement: Supplementary file 1 [file Table1.docx]

| Table S1. Systematic Summary of Mitochondrial Transfer Events in Cancer | | | | | | | | | | |
| --- | --- | --- | --- | --- | --- | --- | --- | --- | --- | --- |
| **No.** | **Donor Cell** | **Recipient Cell** | **Cancer Type / Model** | **Transferred Cargo** | **Transfer**  **Route** | **Experimental Model** | **Detection Method** | **Biological Outcome** | **Level of Evidence** | **Reference** |
| 1* | Neurons | Cancer cells | Breast cancer (4T1) | Intact functional mitochondria | TNTs (direct contact) | In vitro co-culture + in vivo mouse model | MitoTracker staining + flow cytometry + TEM | Enhanced survival under shear stress and oxidative stress | High | Hoover et al., 2025(1) |
| 2* | Platelets | Breast cancer cells | Breast cancer | Intact mitochondria (microparticles) | EVs (microparticles) | In vitro co-culture + in vivo mouse model | MitoTracker staining + confocal microscopy + flow cytometry | Increased ATP, invasive migratory phenotype | High | Veilleux et al., 2024(2) |
| 3* | Platelets | Chronic lymphocytic leukemia cells | Chronic lymphocytic leukemia (CLL) | Intact mitochondria (microparticles) | EVs (microparticles) | In vitro co-culture | MitoTracker staining + confocal microscopy + ATP assay | Increased ATP, enhanced malignant phenotype | Medium | Gharib et al., 2023(3) |
| 4* | Adipose-derived stem cel | Cancer cells | Multiple cancer types | Intact mitochondria | TNTs + gap junctions | In vitro co-culture | MitoTracker staining + confocal microscopy | Metabolic support | Medium | Rehman et al., 2024(4) |
| 5* | Astrocytes | Glioblastoma cells | Glioblastoma | Intact mitochondria | TNTs (GAP43-dependent) | In vitro co-culture + in vivo mouse model | MitoTracker staining + confocal microscopy + in vivo imaging | Enhanced tumorigenicity | High | Watson et al., 2023(5) |
| 6* | Hepatic stellate cells (HSCs) | Hepatocellular carcinoma(HCC) cells | HCC | Intact functional mitochondria | TNTs (Miro1-mediated) | In vitro co-culture + in vivo mouse model | MitoTracker staining + confocal microscopy + TEM | Promoted HCC progression; damaged mitochondria cleared by macrophages via EVs | High | Zhang et al., 2026(6) |
| 7* | Neutrophils | THY1+ cancer stem cells (CSCs) | Pan-cancer (CSCs) | Damaged mitochondria (mtROS-rich) | migrasome release + macropinocytosis | In vitro co-culture + in vivo mouse model | MitoSOX staining + confocal microscopy + flow cytometry | Pseudohypoxic state, metabolic plasticity, anti-apoptotic capacity | High | Wan et al., 2026(7) |
| 8* | Highly metastatic HCC cells | Low-metastatic HCC cells | HCC | Intact mitochondria + mtDNA | TNTs (HMGB1/RHOT1/RAC1) | In vitro co-culture | MitoTracker staining + confocal microscopy + TEM + Western blot | Significantly enhanced migratory and invasive capability | High | Jing et al., 2024(8) |
| 9* | Highly invasive bladder cancer cells (T24) | Low-invasive bladder cancer cells (RT4) | Bladder cancer | Intact mitochondria | TNTs | In vitro co-culture | MitoTracker staining + confocal microscopy + Transwell invasion assay | Enhanced invasive phenotype | Medium | Lu et al., 2017(9) |
| 10* | Breast cancer cells (PINK1-mediated) | Recipient breast cancer cells | Breast cancer | mtDNA (EV-packaged) | EVs (PINK1-mediated packaging) | In vitro co-culture + in vivo mouse model | mtDNA qPCR + TEM + confocal microscopy | Driven migratory and invasive phenotypes | High | Rabas et al., 2021(10) |
| 11* | CD8+ T cells | Tumor cells | Pan-cancer (lymph node metastasis) | Intact mitochondria + mutated mtDNA + USP30 | TNTs (active hijacking) | In vitro co-culture + in vivo mouse model | MitoTracker staining + confocal microscopy + single-cell sequencing + Western blot | T-cell effector dysfunction and memory formation defects, immune evasion | High | Ikeda et al., 2025(11) |
| 12* | CD8+ T cells | Tumor cells | Pan-cancer (immune evasion) | Intact mitochondria | TNTs (active hijacking) | In vitro co-culture + in vivo mouse model | MitoTracker staining + confocal microscopy + flow cytometry + in vivo imaging | Type I interferon response activation, immune evasion program, lymph node metastasis | High | Terasaki et al., 2026(12) |
| 13 | Stromal cells | Tumor cells | Pan-cancer | Intact mitochondria (acquired) | TNTs (active hijacking) | In vitro co-culture | MitoTracker staining + confocal microscopy + fusion assay | Failed to fuse with cancer cell mitochondria; no signaling activation; no metastasis promotion | High | Terasaki et al., 2026(12) |
| 14 | Osteocytes | Tumor cells | Bone metastasis-related tumors | Intact mitochondria (passive uptake) | Passive acquisition | In vitro co-culture + in vivo mouse model | MitoTracker staining + confocal microscopy + in vivo imaging | STING-dependent antitumor immunity | High | Zhou et al., 2024(13) |
| 15* | Senescent tumor cells | PMN-MDSCs | Pan-cancer (immunosuppression) | mtDNA (EV-encapsulated) | EVs | In vitro co-culture + in vivo mouse model | mtDNA qPCR + flow cytometry + Western blot | Enhanced PMN-MDSC immunosuppressive activity | High | Lai et al., 2025(14) |
| 16* | Endothelial cells (ECs) | Melanoma cells | Melanoma | Intact mitochondria | TNTs | In vitro co-culture + in vivo mouse model | MitoTracker staining + confocal microscopy + flow cytometry + in vivo imaging | M1 to M2 macrophage polarization, immunosuppressive microenvironment | Medium | Kuo et al., 2024(15) |
| 17 | Mesenchymal stem cells (MSCs) | CD4+ T cells (Th17) | Multiple sclerosis (non-cancer) | Intact mitochondria | TNTs | In vitro co-culture (EAE model) | MitoTracker staining + confocal microscopy + flow cytometry | Th17 to Treg conversion, IL-17 suppression | Medium | Hu et al., 2024(16) |
| 18 | MSCs | CD4+ T cells | Inflammation model (non-cancer) | Intact mitochondria | TNTs | In vitro co-culture + in vivo mouse model | MitoTracker staining + confocal microscopy + flow cytometry | Treg differentiation, inflammatory response limitation | Medium | Court et al., 2020(17) |
| 19* | Tumor cells | CD8+ T cells | Melanoma | Mutated mtDNA, mitochondria, USP30 | TNTs & EVs (active hijacking) | In vitro co-culture + in vivo mouse model | mtDNA sequencing + single-cell RNA-seq (MERCI) + functional assays | T-cell dysfunction, impaired antitumor immunity, metabolically empowered cancer cells | High | Ikeda et al., 2025(11) |
| 20* | T cells (NK, CD3+/CD8+) | Breast cancer cells | Breast cancer (MDA-MB-231, 4T1) | Intact mitochondria | TNTs | In vitro co-culture + in vivo mouse model | Single-cell RNA-seq (MERCI) + MitoTracker + functional metabolic assays | Metabolically empowered cancer cells, depleted immune cells | High | Zhang et al., 2023(18) |
| 21* | Tumor cells | Cancer-associated fibroblasts (CAFs) | Skin cancer | Intact mitochondria | TNTs (Miro2-mediated) | In vitro co-culture + in vivo mouse model | MitoTracker staining + confocal microscopy + TEM + metabolomics | CAF phenotype acquisition, upregulated OXPHOS, increased ATP production | High | Cangkrama et al., 2025(19) |
| 22* | Tumor cells (B-ALL) | Mesenchymal stromal cells (MSCs) | B-cell acute lymphoblastic leukemia | Mitochondrial dsRNA | EVs | In vitro co-culture | dsRNA detection + confocal microscopy + metabolomics | Driven CAF differentiation | Medium | Burt et al., 2024(20) |
| 23* | CAFs | Triple-negative breast cancer cells | Triple-negative breast cancer | Intact mitochondria | TNTs | In vitro co-culture + in vivo mouse model | MitoTracker staining + confocal microscopy + 3D migration assay + in vivo imaging | Increased mitochondrial ATP production, enhanced 3D migration capacity | High | Goliwas et al., 2023(21) |
| 24* | CAFs | Prostate cancer cells | Prostate cancer | Intact mitochondria | Cell bridges | In vitro co-culture + in vivo mouse model | MitoTracker staining + confocal microscopy + in vivo imaging | Promoted malignant progression | High | Ippolito et al., 2019(22) |
| 25* | CAFs | Adenoid cystic carcinoma cells | Adenoid cystic carcinoma | Intact mitochondria (cell fusion) | TNTs + cell fusion (TMEM16F) | In vitro co-culture + in vivo mouse model | MitoTracker staining + confocal microscopy + cell fusion detection | Restored metabolic function, enhanced malignancy | High | Qiao et al., 2024(23) |
| 26* | Inducible CAFs (iCAFs) | Lung cancer cells | Lung cancer | mtDNA | Conditioned medium | In vitro co-culture + in vivo mouse model | mtDNA qPCR + confocal microscopy + TEM | Restored mitochondrial function, enhanced oxidative stress resistance, promoted metastasis | High | Zhou et al., 2024(24) |
| 27* | Tumor cells | Normal colonic epithelial cells (CECs) | Colorectal cancer (pre-metastatic niche) | Circular mtDNA (EVs) | EVs | In vitro co-culture + in vivo mouse model | mtDNA sequencing + confocal microscopy + TEM + in vivo imaging | Upregulated TGF-beta1, RelA nuclear translocation, pre-metastatic niche formation | High | Guan et al., 2024(25) |
| 28* | Tumor cells | Macrophages | HCC | Damaged mitochondria (EVs) | EVs | In vitro co-culture + in vivo mouse model | MitoTracker staining + confocal microscopy + flow cytometry | Macrophage clearance of damaged mitochondria | High | Zhang et al., 2026(6) |

**Table Legend and Definitions**

1. Asterisk (*) in the "No." column:

* indicates that the mitochondrial transfer event has a pro-tumor effect

Entries without * may represent anti-tumor effects, therapeutic applications, or neutral/undetermined biological outcomes.

2. Level of Evidence definitions:

High: Studies published in top-tier journals (Nature, Cell, Science, Immunity, Cell Metabolism, PNAS, etc.) with both in vitro and in vivo validation, and/or single-cell resolution analysis.

Medium: Studies published in specialized journals with either in vitro or in vivo validation, or extrapolated from non-cancer disease models.

3. Table organization:

This table includes evidence from Manuscript Section 4 only

**Reference**

1.	Hoover G, Gilbert S, Curley O, Obellianne C, Lin MT, Hixson W, et al. Nerve-to-cancer transfer of mitochondria during cancer metastasis. Nature. 2025;644(8075):252-62. 10.1038/s41586-025-09176-8

2.	Veilleux V, Pichaud N, Boudreau LH, Robichaud GA. Mitochondria Transfer by Platelet-Derived Microparticles Regulates Breast Cancer Bioenergetic States and Malignant Features. Mol Cancer Res. 2024;22(3):268-81. 10.1158/1541-7786.Mcr-23-0329

3.	Gharib E, Veilleux V, Boudreau LH, Pichaud N, Robichaud GA. Platelet-derived microparticles provoke chronic lymphocytic leukemia malignancy through metabolic reprogramming. Front Immunol. 2023;14:1207631. 10.3389/fimmu.2023.1207631

4.	Rehman A, Marigliano M, Torsiello M, La Noce M, Papaccio G, Tirino V, et al. Adipose Stem Cells and Their Interplay with Cancer Cells and Mitochondrial Reservoir: A New Promising Target. Cancers (Basel). 2024;16(15). 10.3390/cancers16152769

5.	Watson DC, Bayik D, Storevik S, Moreino SS, Sprowls SA, Han J, et al. GAP43-dependent mitochondria transfer from astrocytes enhances glioblastoma tumorigenicity. Nat Cancer. 2023;4(5):648-64. 10.1038/s43018-023-00556-5

6.	Zhang L, Ren C, He M, Chen X, Liu H, Liu Y, et al. Mitochondrial transfer in the HSC-HCC-macrophage network shapes hepatocellular carcinoma progression. Proc Natl Acad Sci U S A. 2026;123(6):e2512592123. 10.1073/pnas.2512592123

7.	Wan WH, Li PL, Cao WJ, Li ZX, Xin YQ, Wang JC, et al. THY1(+) cancer stem cells drive metastasis through a pseudohypoxic state shaped by neutrophil-derived mitochondria. Nat Cell Biol. 2026. 10.1038/s41556-026-01876-1

8.	Jing M, Xiong X, Mao X, Song Q, Zhang L, Ouyang Y, et al. HMGB1 promotes mitochondrial transfer between hepatocellular carcinoma cells through RHOT1 and RAC1 under hypoxia. Cell Death Dis. 2024;15(2):155. 10.1038/s41419-024-06536-6

9.	Lu J, Zheng X, Li F, Yu Y, Chen Z, Liu Z, et al. Tunneling nanotubes promote intercellular mitochondria transfer followed by increased invasiveness in bladder cancer cells. Oncotarget. 2017;8(9):15539-52. 10.18632/oncotarget.14695

10.	Rabas N, Palmer S, Mitchell L, Ismail S, Gohlke A, Riley JS, et al. PINK1 drives production of mtDNA-containing extracellular vesicles to promote invasiveness. J Cell Biol. 2021;220(12). 10.1083/jcb.202006049

11.	Ikeda H, Kawase K, Nishi T, Watanabe T, Takenaga K, Inozume T, et al. Immune evasion through mitochondrial transfer in the tumour microenvironment. Nature. 2025;638(8049):225-36. 10.1038/s41586-024-08439-0

12.	Terasaki A, Bhatnagar K, Weiner AT, Tan Y, Szeifert V, Huang HL, et al. Mitochondrial transfer from immune to tumor cells enables lymph node metastasis. Cell Metab. 2026;38(2):388-98.e7. 10.1016/j.cmet.2025.12.014

13.	Zhou H, Zhang W, Li H, Xu F, Yinwang E, Xue Y, et al. Osteocyte mitochondria inhibit tumor development via STING-dependent antitumor immunity. Sci Adv. 2024;10(3):eadi4298. 10.1126/sciadv.adi4298

14.	Lai P, Liu L, Bancaro N, Troiani M, Calì B, Li Y, et al. Mitochondrial DNA released by senescent tumor cells enhances PMN-MDSC-driven immunosuppression through the cGAS-STING pathway. Immunity. 2025;58(4):811-25.e7. 10.1016/j.immuni.2025.03.005

15.	Kuo FC, Tsai HY, Cheng BL, Tsai KJ, Chen PC, Huang YB, et al. Endothelial Mitochondria Transfer to Melanoma Induces M2-Type Macrophage Polarization and Promotes Tumor Growth by the Nrf2/HO-1-Mediated Pathway. Int J Mol Sci. 2024;25(3). 10.3390/ijms25031857

16.	Hu H, Li H, Li R, Liu P, Liu H. Re-establishing immune tolerance in multiple sclerosis: focusing on novel mechanisms of mesenchymal stem cell regulation of Th17/Treg balance. J Transl Med. 2024;22(1):663. 10.1186/s12967-024-05450-x

17.	Court AC, Le-Gatt A, Luz-Crawford P, Parra E, Aliaga-Tobar V, Bátiz LF, et al. Mitochondrial transfer from MSCs to T cells induces Treg differentiation and restricts inflammatory response. EMBO Rep. 2020;21(2):e48052. 10.15252/embr.201948052

18.	Zhang H, Yu X, Ye J, Li H, Hu J, Tan Y, et al. Systematic investigation of mitochondrial transfer between cancer cells and T cells at single-cell resolution. Cancer Cell. 2023;41(10):1788-802.e10. 10.1016/j.ccell.2023.09.003

19.	Chang X, Yao Y, Zhang M, Sun C, Zhang Q. Spatiotemporal dynamics of the pre-metastatic niche: remodeling of lung microenvironment before breast cancer metastasis and targeted intervention strategies. Biochim Biophys Acta Rev Cancer. 2025;1880(4):189360. 10.1016/j.bbcan.2025.189360

20.	Burt RJ, Dey A, Akarca A, Allen H, Amerikanou R, Atkinson S, et al. Mitochondrial dsRNA from B-ALL cells stimulates mesenchymal stromal cells to become cancer-associated fibroblasts. Blood Adv. 2024;8(21):5696-709. 10.1182/bloodadvances.2023012077

21.	Goliwas KF, Libring S, Berestesky E, Gholizadeh S, Schwager SC, Frost AR, et al. Mitochondrial transfer from cancer-associated fibroblasts increases migration in aggressive breast cancer. J Cell Sci. 2023;136(14). 10.1242/jcs.260419

22.	Ippolito L, Morandi A, Taddei ML, Parri M, Comito G, Iscaro A, et al. Cancer-associated fibroblasts promote prostate cancer malignancy via metabolic rewiring and mitochondrial transfer. Oncogene. 2019;38(27):5339-55. 10.1038/s41388-019-0805-7

23.	Qiao X, Huang N, Meng W, Liu Y, Li J, Li C, et al. Beyond mitochondrial transfer, cell fusion rescues metabolic dysfunction and boosts malignancy in adenoid cystic carcinoma. Cell Rep. 2024;43(9):114652. 10.1016/j.celrep.2024.114652

24.	Zhou Z, Qu C, Zhou P, Zhou Q, Li D, Wu X, et al. Extracellular vesicles activated cancer-associated fibroblasts promote lung cancer metastasis through mitophagy and mtDNA transfer. J Exp Clin Cancer Res. 2024;43(1):158. 10.1186/s13046-024-03077-w

25.	Guan B, Liu Y, Xie B, Zhao S, Yalikun A, Chen W, et al. Mitochondrial genome transfer drives metabolic reprogramming in adjacent colonic epithelial cells promoting TGFβ1-mediated tumor progression. Nat Commun. 2024;15(1):3653. 10.1038/s41467-024-48100-y
